# Rare, Yet Targetable: New Perspectives on Ampullary Carcinomas

**DOI:** 10.3390/ijms27031597

**Published:** 2026-02-06

**Authors:** James Gutmans, Alex Friedlaender, Hiba Mechahougui

**Affiliations:** 1 Oncology Department, Geneva University Hospital (HUG), 1205 Geneva, Switzerland; 2 Clinique Générale Beaulieu, 1206 Geneva, Switzerland

**Keywords:** ampullary carcinoma, intestinal subtype, pancreatobiliary subtype, precision oncology, next-generation sequencing, liquid biopsy, rare cancer

## Abstract

Ampullary carcinoma (AC) is a rare gastrointestinal malignancy with dual intestinal and pancreatobiliary differentiation, complicating diagnosis, staging, and treatment. This review synthesizes current epidemiology, pathology, and multi-omic data to outline a pragmatic care pathway: lineage-first at presentation, mutation-fast at progression. Histology remains the primary classifier: the intestinal subtype generally aligns with colorectal regimens, whereas pancreatobiliary and mixed subtypes favor pancreaticobiliary therapy. In selected fit patients, modified FOLFIRINOX may address mixed phenotypes. Next-generation sequencing adds precision by identifying therapeutically relevant alterations, including *ERBB2*/*HER2* amplifications, MSI-high/dMMR, *BRAF* V600E, and rare *NTRK* or *RET* fusions, while *KRAS* mutations are enriched in pancreatobiliary tumors. We recommend early application of a rapid-core panel (*KRAS*/*BRAF*, MSI/dMMR, *ERBB2*/*HER2*, RNA-based fusions) to capture high-impact targets, followed by comprehensive profiling at first progression. Liquid biopsy, plasma circulating tumor DNA (ctDNA), or bile-derived DNA may complement tissue and help identify the dominant lineage. Research priorities include ampulla-enriched umbrella trials, explicit AC subcohorts in tissue-agnostic studies, and ctDNA-informed endpoints. This lineage-first, mutation-fast paradigm supports precision care and evidence generation in AC.

## 1. Introduction

Ampullary carcinoma (AC) is a rare and distinct malignancy arising at the ductal confluence of the ampulla of Vater, where the pancreatic and common bile ducts enter the second portion of the duodenum [1,2]. It accounts for approximately 0.2% of gastrointestinal cancers and 6% of periampullary tumors, with an estimated global incidence of ~6 cases per million person-years. AC typically presents in the sixth to seventh decades of life, shows a modest male predominance (≈1.5:1), and displays geographic variability, with higher reported incidence in Western compared with Asian populations, likely reflecting differences in environmental, dietary, and genetic factors [3,4,5,6].

Historically managed alongside pancreatic and biliary tract cancers, AC warrants separate consideration. Obstructive jaundice, abdominal pain, nausea, weight loss, fever, back pain, and occasional pancreatitis are common, with pooled series reporting jaundice in 80% [7], and fever and abdominal pain in 45% of patients [8]. Contemporary imaging and endoscopy, ERCP, EUS, and cross-sectional CT/MRI have improved staging and tissue acquisition, yet ambiguous appearances at the papilla still complicate diagnosis [6,9].

Increasing recognition of AC’s molecular diversity has opened therapeutic windows: next-generation sequencing reveals actionable alterations in over half of cases, including *ERBB2*/*HER2* amplification/mutation, MSI-H/dMMR, and homologous recombination deficiency, findings that are already informing genotype-directed care [1,3,10].

In this review, we summarize epidemiologic, histologic, and molecular data in AC with the explicit goal of moving beyond descriptive subclassification toward a clinically operational framework. We integrate histologic lineage with contemporary genomic profiling to propose a pragmatic, stepwise treatment paradigm that reconciles lineage-guided chemotherapy with biomarker-driven precision oncology. In doing so, we identify where extrapolation from pancreatic, biliary, and colorectal cancers is justified, where it fails, and where AC-specific trial design is urgently needed, thereby defining priorities for future translational and clinical research.

### 1.1. Histological Subtypes

Histology remains the most practical stratifier in AC, with reproducible prognostic and therapeutic implications across three lineages: pancreatobiliary, intestinal, and mixed histology. Subtyping is based on the dominant invasive component. On pre-resection biopsies, limited sampling can obscure the prevailing pattern, and ancillary immunohistochemistry is often needed to resolve ambiguous cases [6,9]. Importantly, histotype does more than label morphology; it aligns with patterns of spread, survival, and the selection of colorectal-style versus pancreaticobiliary-style systemic therapies [9,11].

#### 1.1.1. Pancreatobiliary Subtype

The most prevalent lineage, accounting for 50–60% of AC, the pancreatobiliary subtype, has a pancreatic/biliary epithelium [12,13]. Histologically, it shows complex, tightly packed glandular structures with marked cytologic atypia, a prominent desmoplastic stroma, and frequent perineural invasion, consistent across comparative series of ampullary histotypes [14]. Clinically, this lineage exhibits more aggressive behavior, higher nodal and distant spread and poorer outcomes in subtype-stratified analyses [15]. Survival outcomes are poor, with median overall survival of approximately 40–45 months and a 5-year survival rate of around 8–15% in subtype-stratified surgical series and retrospective cohort analyses, reflecting both aggressive tumor biology and relative resistance to conventional cytotoxic therapy compared with the intestinal subtype [15,16].

#### 1.1.2. Intestinal Subtype

Representing 25–35% of cases, the intestinal subtype mirrors colorectal adenocarcinoma [4]. The invasive component typically forms well-organized glands lined by columnar cells with pseudostratified nuclei. Goblet cell differentiation and extracellular mucin are common [17]. This phenotype is associated with a less aggressive clinical course, lower nodal involvement and metastatic potential, and consistently superior survival versus the pancreatobiliary subtype [15]. In resected cohorts and pooled retrospective surgical series, median overall survival approaches 70–80 months, with reported 5-year survival rates of around 50–60%, consistently better than the pancreatobiliary subtype [15]. Therapeutically, the intestinal subtype often benefits from regimens borrowed from colorectal cancer (e.g., FOLFOX or CAPOX), with multiple series reporting notable activity in this subgroup [8,18,19].

#### 1.1.3. Mixed Subtype

The mixed subtype accounts for 10–20% of AC and blends intestinal and pancreatobiliary features to varying degrees [6]. Its heterogeneity creates diagnostic and prognostic ambiguity, including variable gland formation, cytologic atypia, and stromal response within the same tumor [4,9]. Available data suggest outcomes tend to approximate those of the pancreatobiliary subtype.

When morphology is equivocal, limited immunopanels (for example, CK7/CK20, CDX2, MUC1/MUC2) can aid in the diagnosis. Reporting mixed histology is preferable to an improper diagnosis when tissue is scant, given the implications for systemic therapy [6,9].

### 1.2. Liquid Biopsy: Promise, Constraints, and a Realistic Near-Term Role in AC

**Figure 1:**
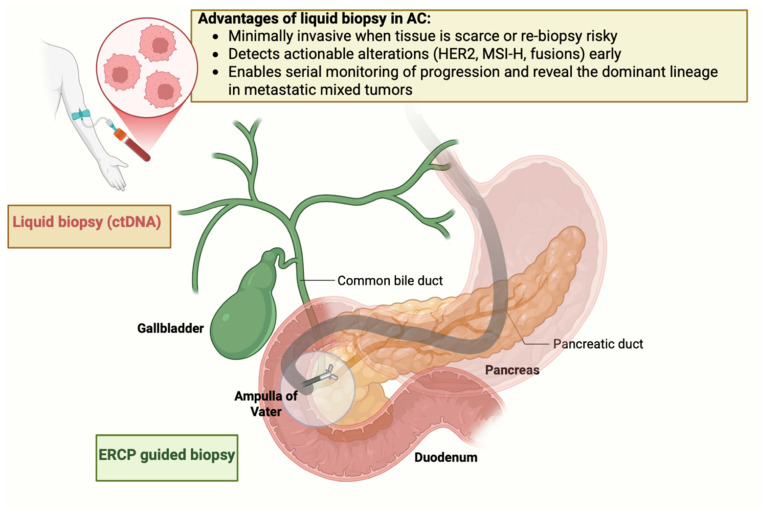
Liquid biopsy and ERCP-guided sampling: complementary tools in ampullary carcinoma.

Obtaining and repeating tissue biopsies in AC is often challenging. Diagnostic sampling frequently relies on small endoscopic biopsies of a friable papilla, where intraductal spread, submucosal invasion, post-sphincterotomy fibrosis, and superimposed inflammation can all limit yield. Repeat biopsies at progression are constrained by anatomy and the risks of Endoscopic retrograde cholangiopancreatography (ERCP)/Endoscopic ultrasound (EUS)-guided procedures (pancreatitis, cholangitis, bleeding). Liquid biopsy, which is the analysis of circulating tumor DNA (ctDNA) and cell-free DNA (cfDNA), exosomal nucleic acids, and circulating tumor-associated cells, offers a minimally invasive means to establish or confirm actionable alterations when tissue is scant and to serially monitor molecular evolution [20] (Figure 1).

Although prospective AC-specific trials are lacking, several informative real-world clinical studies illustrate how liquid biopsy approaches may influence management in selected contexts. In a study that included an ampullary subset, bile-derived ctDNA obtained during ERCP was interrogated for *KRAS* hotspots by digital droplet PCR. In this subgroup, bile ctDNA demonstrated high concordance with matched formalin-fixed and paraffin-embedded tumor and outperformed plasma ctDNA for both sensitivity and mutation–tissue agreement. Detection of *KRAS* in bile was associated with worse survival, hinting at prognostic utility [21,22]. These data support a biologically intuitive concept: bile, in direct contact with the tumor epithelium, can be a high-yield analyte in AC, but the approach is invasive, procedure-dependent, and not readily suited to frequent longitudinal sampling outside preplanned ERCPs.

Blood-based, multi-analyte assays are even less studied in AC but have proof-of-principle signals. A periampullary adenocarcinoma case report combined cell-free DNA (cfDNA) genotyping with exosomal microRNA profiling, identifying KRAS p.G12D (supporting pancreatobiliary lineage) and a signature consistent with gemcitabine resistance but cisplatin sensitivity. Switching therapy produced a rapid response, with CA19-9 falling from 3290 U/mL to 90 U/mL within eight weeks [23].

Currently, liquid biopsy is best positioned as a complement to tissue, not a replacement. At baseline, when tissue is insufficient or non-representative, it can help identify core biomarkers that influence early management (MSI/dMMR, *ERBB2*/*HER2* status, rare fusions such as NTRK/RET, and KRAS/BRAF class) [20]. At first radiographic progression, repeat liquid sampling can help detect emergent targets for later-line therapy or trial referral, acknowledging that AC-specific resistance atlases are not yet available.

In tumors reported as mixed (morphology and/or IHC), serial ctDNA can help infer which lineage is driving dissemination. Dominance of KRAS hotspot mutations with a pancreatobiliary-type molecular profile would support prioritizing pancreatobiliary backbones, whereas RAS wild-type status with alterations characteristic of intestinal/WNT biology (for example, APC/RNF43) may justify favoring colorectal backbones (FOLFOX/CAPOX/FOLFIRI) and, in later lines, considering tissue-agnostic targets (HER2, MSI-H/dMMR, NTRK/RET) if present [9]. These inferences should be integrated with imaging and pathology, and treated as hypothesis-generating until validated in AC-specific cohorts.

For alterations most relevant to AC (*KRAS*, *BRAF* V600E, *ERBB2*, kinase fusions), resistance mechanisms are largely inferred from pancreatic ductal adenocarcinoma (PDAC) and metastatic colorectal cancer (mCRC), MAPK reactivation via secondary RAS/RAF/MAPK mutations and bypass tyrosine kinase signaling (for example, *MET*/*ERBB*). These events have not been systematically mapped in AC, and routine liquid-guided resistance management is not recommended at present outside trials or highly selected cases [20]. The bile-ctDNA *KRAS* signal described above is hypothesis-generating for prognosis, but not yet a validated trigger for treatment change [21,22].

Pre-analytical variables (low tumor fraction in plasma, biliary inflammation), assay breadth (copy-number and fusions often requiring RNA capture), and analyte choice (bile versus plasma) all influence performance. Bile-ctDNA analysis requires ERCP expertise and standardized handling, limiting scalability. What is needed are prospective, AC-enriched studies that pair tissue and liquid assays at baseline and progression, pre-specify decision points, and test whether liquid-guided interventions improve outcomes (response, time-to-next-treatment, survival).

Liquid biopsy in AC is promising but unproven. Bile-derived ctDNA can improve mutation detection when ERCP is already indicated, and blood-based assays can occasionally inform therapy when tissue is unobtainable. In mixed-lineage disease, ctDNA may help identify the metastasizing component and guide which systemic strategy to choose. Outside clinical trials, liquid biopsy should be used selectively and interpreted alongside histology, imaging, and the genomic context of related pancreatobiliary and intestinal cancers [20,21,22,23].

## 2. Management

### 2.1. Localized Setting

For localized, resectable disease, pancreaticoduodenectomy remains the standard potentially curative approach, ideally in high-volume centers. However, based on national registry-based retrospective cohort data, recurrence rates following R0 resection approach 40–50%, and reported 5-year overall survival ranges from approximately 30 to 35%, underscoring the need for effective perioperative systemic therapy and strict postoperative surveillance [11,24].

Without AC-specific randomized trials, neoadjuvant therapy cannot be considered standard. Aggregated data from duodenal and ampullary studies [25] show variable response rates and lack a consistent survival advantage. When considered for selected high-risk presentations (borderline resectability, bulky nodes, involved margins), decisions should be individualized in multidisciplinary boards at high-volume centers, and ideally embedded in prospective protocols [11,26,27].

Following R0/R1 pancreaticoduodenectomy, adjuvant systemic chemotherapy is widely regarded as part of the standard of care in AC, despite the absence of randomized trials dedicated exclusively to AC. The rationale derives chiefly from ESPAC-3, which enrolled periampullary cancers and AC, and, in adjusted analyses, demonstrated improved overall survival with postoperative chemotherapy (5-fluorouracil/folinic acid or gemcitabine) versus observation [28].

Regimen selection is aligned with histology. Intestinal-type tumors are generally managed with colorectal backbones, 5-fluorouracil, FOLFOX, CAPOX, or capecitabine, whereas pancreatobiliary and mixed subtypes typically follow pancreatic/hepatobiliary protocols (gemcitabine-based combinations, FOLFOX/CAPOX, capecitabine), with consideration of modified FOLFIRINOX in very fit patients after multidisciplinary discussion. These principles are also reflected in French Intergroup Clinical Practice Guidelines, which recommend monotherapy (gemcitabine or 5-FU/capecitabine) after resection and, in the presence of high-risk features (pT3/T4, pN+, R1, poor differentiation, pancreatobiliary or mixed histology), six months of polychemotherapy stratified by histologic subtype [9].

The role of adjuvant chemoradiation remains uncertain at a population level. Multicenter series suggest a potential benefit in high-risk settings, particularly node-positive or margin-positive disease, but results are heterogeneous and should be weighed cautiously [28].

### 2.2. Metastatic Setting

#### 2.2.1. First-Line Chemotherapy

For patients with unresectable or metastatic AC, first-line systemic therapy should be anchored to histology rather than genotype. The intestinal subtype is best approached with colorectal backbones such as fluoropyrimidine/capecitabine-oxaliplatin (for example, FOLFOX or CAPOX), whereas pancreatobiliary and mixed histologies are managed with pancreaticobiliary regimens, including gemcitabine-cisplatin or gemcitabine-nab-paclitaxel. In carefully selected, physiologically fit patients, FOLFIRINOX may be considered by extrapolation from pancreatic ductal adenocarcinoma. Patients with poor performance status are generally offered monotherapy with gemcitabine, capecitabine, or 5-fluorouracil [11].

#### 2.2.2. Genomic Characterization and Molecular Targets

**Figure 2:**
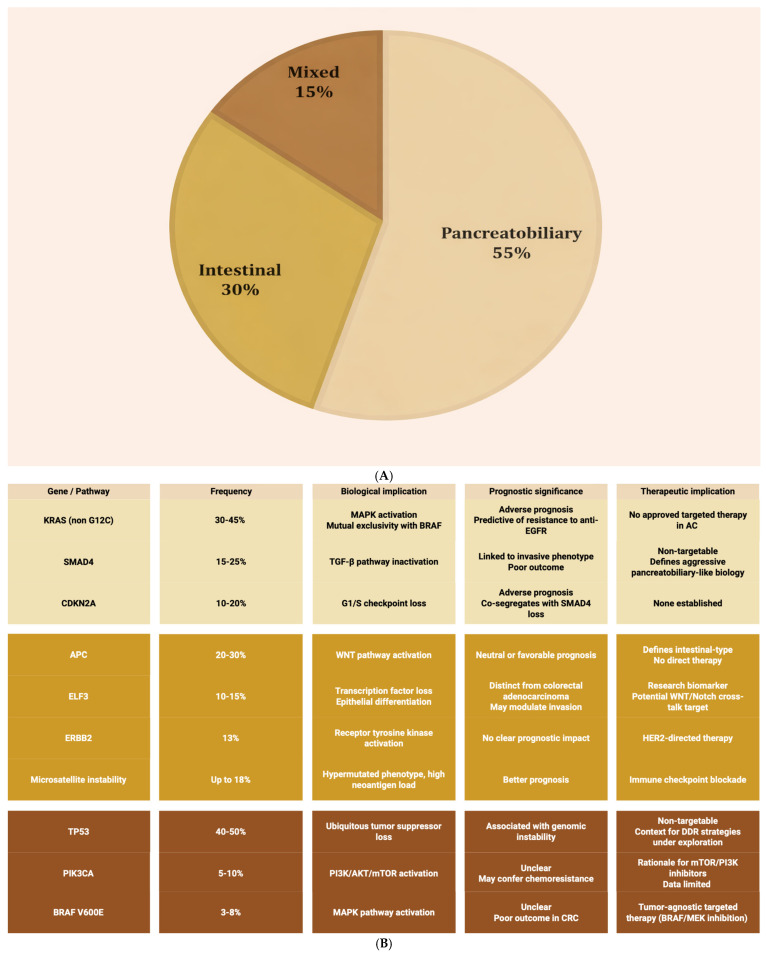
Histotype and core oncogenic/tumor suppressor events. Panel (**A**) illustrates the relative distribution of ampullary carcinoma histotypes, highlighting the predominance of the pancreatobiliary subtype (~55%), followed by the intestinal subtype (~30%) and mixed histology (~15%). Panel (**B**) summarizes recurrent genomic alterations across ampullary carcinoma, grouped by oncogenic signaling pathways and tumor suppressor mechanisms. Color coding reflects histological subtypes, consistent with Panel (**A**): pancreatobiliary (light beige), intestinal (gold), and mixed (brown), highlighting subtype-enriched molecular patterns. Summary of abbreviations: AKT: protein kinase B; APC: adenomatous polyposis coli; BRAF: v-Raf murine sarcoma viral oncogene homolog B; CDKN2A: cyclin-dependent kinase inhibitor 2A; DDR: DNA damage response; dMMR: deficient mismatch repair; EGFR: epidermal growth factor receptor; ELF3: E74-like ETS transcription factor 3; *ERBB2*/*HER2*: human epidermal growth factor receptor 2; GI: gastrointestinal; KRAS: Kirsten rat sarcoma viral oncogene homolog; MAPK: mitogen-activated protein kinase; mTOR: mechanistic target of rapamycin; MSI-H: microsatellite instability-high; PI3K: phosphoinositide 3-kinase; PIK3CA: phosphatidylinositol-4,5-bisphosphate 3-kinase catalytic subunit alpha; RTK: receptor tyrosine kinase; SMAD4: SMAD family member 4; TP53: tumor protein p53; WNT: wingless-related integration site; WT: wild-type.

Advancements in next-generation sequencing (NGS) have redefined the molecular taxonomy of AC, revealing lineage-linked architectures and a spectrum of therapeutically relevant alterations that differ between intestinal and pancreatobiliary subtypes [1,15]. Across comprehensive series, recurrent mutations involve *KRAS*, *TP53*, *APC*, *SMAD4*, *ELF3*, *PIK3CA*, and *CDKN2A*, underscoring convergent disruption of MAPK signaling, WNT regulation, TGF-β pathways, and cell-cycle control [3,29,30,31,32,33,34,35,36,37] (Figure 2).

- Lineage-associated architectures

*KRAS* mutations are particularly enriched in the pancreatobiliary lineage, occurring in approximately 30–45% of cases and associating with adverse prognosis and lack of benefit from anti-EGFR therapies. This mirrors resistance biology observed in pancreatic and *RAS*-mutant colorectal cancers [30,38]. By contrast, the intestinal subtype frequently harbors *APC* and *TP53* alterations, consistent with a colorectal-like molecular signature and supporting the application of colorectal treatment paradigms in this subtype [31].

- Core tumor suppressors and pathway nodes

*SMAD4* loss and *CDKN2A* inactivation are consistently linked to inferior outcomes, reflecting disruption of TGF-β signaling and G1/S checkpoint control, respectively. These alterations are associated with more invasive biology and higher metastatic propensity [33,36]. The PI3K/AKT/mTOR axis is occasionally altered, most notably via *PIK3CA* mutations, providing a mechanistic rationale for pathway inhibition in selected cases, though prospective evidence in AC remains limited [36,39]. *ELF3* emerges as a recurrent driver in multi-omic studies and, together with *APC*/*RNF43* alterations, anchors a WNT-addicted subset that aligns with the intestinal phenotype [34].

- Clinically actionable biomarkers

A clinically meaningful subset of AC carries alterations amenable to targeted or tissue-agnostic therapies. *ERBB2*/HER2 amplification or overexpression is identified in roughly 13% of cases, particularly within the intestinal subtype, supporting consideration of HER2-directed approaches such as trastuzumab-based combinations or dual blockade [36]. MSI-H or dMMR occurs in a minority (approximately 2–6%) but has significant therapeutic impact, as these tumors are sensitive to PD(L)-1 blockade. Although high tumor mutational burden (TMB) may correlate with increased neoantigen load and thus potential sensitivity to checkpoint inhibition [40,41], it is an inconsistent predictor of response, as many TMB-high tumors lack an inflamed microenvironment or functional antigen presentation. Other rare but actionable events include kinase fusions such as *NTRK*, *RET*, and oncogenic *BRAF* V600E, which enable access to tissue-agnostic targeted agents and genotype-matched trials in later lines [42].

- Implications for testing and sequencing

Taken together, these data reinforce a sequencing paradigm in which first-line therapy is histology-directed, while rapid, comprehensive molecular profiling, preferably including copy-number assessment and RNA fusion analysis, is prioritized at progression to uncover HER2, MSI-H/dMMR, kinase fusions, and other targets that can shape second-line and subsequent strategies [1,39].

### 2.3. Targeted Therapy

Targeted therapy comprises biomarker-driven agents that inhibit defined oncogenic drivers, in contrast to the non-selective cytotoxicity of conventional chemotherapy. In AC, prospective evidence remains sparse and largely derived from basket trials or single-arm phase II studies with small AC subsets (Table 1). Nevertheless, these data delineate which targets are “real” in the clinic and which are extrapolations from related gastrointestinal lineages [2].

#### 2.3.1. What Has Actually Been Tested in Ampullary Carcinoma?

AC data come from three studies. First, MEK inhibition: the Japanese phase IIa trametinib study in gemcitabine-refractory biliary tract cancers enrolled 20 patients, including one (5%) with an ampulla of Vater primary. Although the 12-week non-progression rate was 10% overall and the primary endpoint was not met, one patient experienced prolonged benefit (>120 weeks) alongside *NF1* and *ARID1A* alterations, illustrating how co-mutations can condition MAPK dependence [47]. Second, BRAF targeting: the BEAVER trial (encorafenib and binimetinib) reported a confirmed partial response in an AC harboring *BRAF* D594G among a cohort of non-V600E *BRAF*-mutated solid tumors, supporting occasional sensitivity of “class 3” *BRAF* in combination with MEK inhibition [48,57]. Third, a *BRAF* V600E case report described complete radiographic and endoscopic remission with single-agent vemurafenib in the unresectable pancreatobiliary subtype, hinting at a biology distinct from colorectal cancer with regard to EGFR feedback [58].

#### 2.3.2. HER2

HER2 is a transmembrane tyrosine kinase receptor involved in cell proliferation and survival. While well-characterized in breast and gastric cancers, *ERBB2* overexpression and amplification have also been observed in AC, primarily in the intestinal subtype [3,59,60]. Estimates vary, but *ERBB2*/HER2 amplification is seen in approximately 13% of AC, with amplification more commonly seen in the intestinal histology. Amplification does not appear to affect prognosis [36].

HER2-directed agents, trastuzumab, pertuzumab, and trastuzumab deruxtecan, show meaningful activity across HER2-positive GI malignancies, lending support to their use in AC by extrapolation [61,62,63]. Although AC lacks large, randomized trials, basket studies and real-world series provide signals of benefit. In the phase II, multicenter MyPathway basket trial [64], dual HER2 blockade with trastuzumab plus pertuzumab was evaluated in 39 patients with metastatic biliary tract carcinomas, comprising intrahepatic (*n* = 7), extrahepatic (*n* = 7), gallbladder (*n* = 16), AC (*n* = 5), and site-undesignated (*n* = 4). With a median follow-up of 8.1 months, the overall response rate (ORR) was 23% (9/39; 95% CI, 11–39), disease control rate 51%, median PFS 4.0 months, and median OS 10.9 months. Exploratory analyses suggested a lower benefit in the presence of concurrent *KRAS* mutations [43,64].

In DESTINY-PanTumor02 [44], trastuzumab deruxtecan achieved an ORR of 37.1% with median PFS 6.9 months and OS 13.4 months across HER2-expressing solid tumors. AC was included, but too few to report separately.

Although case-level evidence exists in AC (for example, trastuzumab plus chemotherapy achieving disease control) [45], no HER2-targeted therapy is FDA-approved specifically for AC. Several trials are underway in biliary tract cancers like RC48-ADC [65] or DESTINY-BTC-01 (NCT06467357) that may ultimately refine the evidence base for AC.

#### 2.3.3. RAS-RAF Pathway Targeting

The RAS-RAF-MEK-ERK cascade is a principal effector of receptor tyrosine kinase signaling, and its constitutive activation is a defining engine of epithelial oncogenesis. KRAS functions as a membrane-anchored molecular switch cycling between inactive GDP-bound and active GTP-bound states [66]. Guanine exchange factors (for example, SOS1) load GTP, and GTPase-activating protein (for example, NF1) accelerates hydrolysis. Oncogenic point mutations at codon 12 impair GAP-mediated GTP hydrolysis and stabilize the active conformation, driving persistent engagement of RAF kinases, downstream MEK/ERK phosphorylation, transcriptional programs like MYC, and proliferative/survival phenotypes. Parallel effector arms and ERK-dependent negative feedback loops modulate signal strength, but high RAS activity typically overwhelms these brakes, one reason *KRAS* mutations confer primary resistance to EGFR-directed monoclonal antibodies across gastrointestinal malignancies [67,68]. In AC, *KRAS* mutations are detected in approximately 30–45% of cases, enriched in pancreatobiliary lineage and dominated by p.G12D/p.G12V substitutions; p.G12C is uncommon (0.8–7.1%) [30,69].

- Clinical experience with KRAS-directed therapy.

In CodeBreaK 100, sotorasib achieved an ORR of 21% and disease control of 84% in *KRAS* p.G12C-mutated pancreatic cancer. AC-specific data were not reported [68]. KRYSTAL-1, testing adagrasib across *KRAS* p.G12C-mutated solid tumors, reported an ORR of 35.1% and median PFS of 6.9 months [70]. For the more prevalent non-G12C alleles in AC, investigational approaches include pan-KRAS inhibitors, SHP2 inhibitors, and downstream MEK blockade [71].

- BRAF-targeted therapy.

*BRAF* mutations occur in 3–8% of AC. The ROAR basket trial (dabrafenib and trametinib) demonstrated robust activity in biliary tract cancers with *BRAF* V600E mutations (ORR 53%, median PFS 9.0 months, OS 13.5 months), informing a tumor-agnostic FDA approval relevant to AC when the genotype is present [72]. A single-patient complete response to vemurafenib in unresectable BRAF V600E-mutated AC shows that EGFR-independent MAPK signaling may occasionally be sensitive to BRAF monotherapy, in contrast to colorectal cancer. The histotype was not reported, but it would be particularly interesting if an intestinal subtype responded to BRAF inhibition alone, further highlighting this contrast [58]. The BEAVER study adds a prospective example of response in non-V600E *BRAF* (D594G) AC to encorafenib + binimetinib [48].

#### 2.3.4. NTRK Fusions

Though exceedingly rare, *NTRK* gene fusions (in *NTRK1*, *NTRK2*, or *NTRK3*) represent a “golden ticket” for targeted therapy given the remarkable efficacy of TRK inhibitors. Pooled analyses of larotrectinib (LOXO-TRK-14001/SCOUT/NAVIGATE) report ORR 75–79% with prolonged durability, and entrectinib shows ORR of 57% with intracranial activity across histologies [73,74,75,76]. Although published AC cases are not yet documented, guidelines endorse TRK inhibitors for any AC with an authentic NTRK fusion [11].

#### 2.3.5. RET Fusions

The *RET* gene encodes a receptor tyrosine kinase that transduces growth and survival signals through the RAS/MAPK and PI3K pathways [77]. When *RET* is fused to a partner gene, the resulting chimeric protein becomes constitutively active, independent of ligand binding, leading to uncontrolled cellular proliferation. Although *RET* fusions are rare across most gastrointestinal malignancies (<1% in pancreatic, biliary tract and colorectal cancers), they are clinically relevant due to their high oncogenic potential and sensitivity to targeted inhibition [78].

Selpercatinib is a highly selective RET kinase inhibitor with central nervous system activity. In LIBRETTO-001 [52], among 41 evaluable patients with *RET* fusion, positive advanced solid tumors other than lung and thyroid cancer, including pancreatic, biliary tract, and colorectal cancers, the confirmed ORR was 43%, median duration of response 24.5 months, and median progression-free survival (PFS) 13.2 months. In pancreatic cancer (*n* = 11), ORR was 54.5%. Based on these data, the FDA granted tissue-agnostic approval for selpercatinib [79].

#### 2.3.6. Lineage-Guided Extrapolation When AC-Specific Data Are Absent

In the absence of AC-specific evidence, treatment is guided by analogy, following the dominant histologic lineage. For the pancreatobiliary subtype, first-line management follows distal biliary tract cancer standards (for example, gemcitabine-cisplatin with durvalumab-chemotherapy is now a widely adopted standard after TOPAZ-1 [80] and supportive signals from KEYNOTE-966) [81]. For the intestinal subtype, colorectal backbones (FOLFOX/CAPOX/FOLFIRI) are appropriate. However, despite compelling mCRC data for anti-EGFR therapy in left-sided, *RAS* wild-type disease, there is insufficient evidence to recommend cetuximab or panitumumab broadly in metastatic AC outside of a clinical trial, given biological heterogeneity and the frequent presence of resistance determinants (for example, *KRAS* mutation). For KRAS G12C–mutated tumors, dual EGFR and KRAS inhibition, such as adagrasib combined with cetuximab (ORR 43%) [82], has produced among the most favorable outcomes reported to date in mCRC, but remains extrapolative as patients with AC were not included in these trials. Finally, for HER2-positive intestinal-type tumors, it is reasonable to draw on the HER2-positive mCRC paradigm—trastuzumab plus pertuzumab [83] or trastuzumab plus tucatinib [84], followed by trastuzumab deruxtecan [85]—with the caveat that AC was not included in these studies, necessitating individualized assessment of benefit and risk in each patient.

#### 2.3.7. Microsatellite Instability and Immunotherapy

Microsatellite instability (MSI) results from defective DNA mismatch repair, leading to widespread mutations and frameshift errors within microsatellite regions. It is a hallmark of certain hereditary cancer syndromes, notably Lynch syndrome, and is increasingly tested as a biomarker across gastrointestinal malignancies [86]. In AC, MSI-high is observed in up to 18% of cases, predominantly in the intestinal subtype, and is associated with fewer nodal metastases and improved survival compared with microsatellite stable (MSS) tumors [41]. The elevated neoantigen load renders MSI-high tumors particularly sensitive to PD-1/PD-L1 blockade [87].

In the phase II KEYNOTE-158 trial, pembrolizumab produced an ORR of 34.3% and a median response duration of 22.7 months in previously treated MSI-high/dMMR non-colorectal cancers, forming the basis for FDA approval in unresectable or metastatic MSI-H/dMMR solid tumors [53]. For the intestinal subtype, whose biology closely mirrors that of colorectal cancer, the combination of nivolumab and low-dose ipilimumab from CheckMate 142 is regarded as a preferred option in MSI-H/dMMR disease, achieving an ORR of 69% and durable PFS and OS in treatment-naïve MSI-H/dMMR mCRC [54]. Dostarlimab has also shown clinically meaningful activity across MSI-H/dMMR gastrointestinal malignancies [54].

Beyond MSI-H/dMMR, a high TMB (≥10 mut/Mb) offers an additional, tissue-agnostic route to PD-1 blockade, supported by CheckMate 848, which demonstrated the benefit of nivolumab plus ipilimumab in TMB-high tumors, predominantly colorectal cancers [55].

#### 2.3.8. Epigenetic Alterations in Ampullary Carcinoma

Although the genomic landscape of ampullary carcinoma (AC) has been increasingly characterized, epigenetic alterations remain comparatively underexplored and are not yet incorporated into routine clinical decision-making. Nevertheless, available evidence suggests that epigenetic dysregulation contributes to tumor biology. Aberrant DNA methylation and epigenetic silencing of tumor suppressor and adhesion-related genes—such as CTNNA1, SMARCD1, and MDC1—have been described across hematologic and solid malignancies [88,89,90]. These observations are relevant to AC given the recurrent involvement of chromatin-remodeling genes, particularly components of the SWI/SNF complex, including ARID1A and ARID2, which regulate histone modification and nucleosome positioning and may drive broader epigenomic instability when altered [91].

Limited evidence also points to microRNA dysregulation in periampullary and ampullary tumors, with altered miRNA expression affecting Wnt/β-catenin, PI3K/AKT, and DNA damage response pathways. However, these findings are largely extrapolated from small series or adjacent gastrointestinal malignancies rather than AC-specific cohorts [92,93]. To date, no characteristic non-canonical DNA structures, such as G-quadruplexes or R-loops, have been systematically described in AC, representing a clear knowledge gap despite their emerging relevance in other gastrointestinal cancers.

## 3. Discussion and Conclusions

In conclusion, while the understanding of histologic subtypes and recurrent genomic alterations in AC has substantially improved, their integration into routine therapeutic decision-making is not yet standard. In this review, we summarize current clinical and translational evidence to identify clinically actionable inflection points, delineate the limits of extrapolation from related gastrointestinal malignancies, and highlight areas where AC-specific investigation is most urgently needed. This framework provides a pragmatic basis for aligning biologic insight with treatment selection in a disease that remains underrepresented in prospective trials.

In this context, histologic subtyping remains the most reliable determinant of natural history and cytotoxic sensitivity. Intestinal disease aligns with colorectal backbones, whereas pancreatobiliary and mixed disease align with pancreaticobiliary regimens. Next-generation sequencing adds options (MSI-H/dMMR, *ERBB2*/*HER2*, BRAF V600E, and rare fusions) that refine therapy after the first line.

Therefore, framing AC as simply “at the crossroads” of intestinal and pancreatobiliary lineages is clinically useful but biologically incomplete. Comparative genomic studies show that the intestinal subtype only partially mirrors right-sided colorectal cancer: APC/TP53 disruption is shared but ELF3 loss, a recurrent alteration in AC, is uncommon in colorectal cohorts [91]. Conversely, the pancreatobiliary subtype shares KRAS/SMAD4/CDKN2A axes with pancreatic ductal adenocarcinoma and distal cholangiocarcinoma, yet retains a mixed identity with differences in co-mutations [69,94] and, notably, in outcomes after resection [93]. Duodenal adenocarcinoma (DA) provides another instructive comparator: MSI rates are typically higher than in AC, and the distribution of WNT/PI3K/BRAF events differs by small-bowel subsite, underscoring that the intestinal subtype is not synonymous with either right colon or DA [16]. These contrasts illustrate that AC possesses a distinct mutational landscape and tumor ecology at the papillary junction that is more than the sum of its lineages.

Prognostically, AC, including the pancreatobiliary subtype, outperforms PDAC and often distal cholangiocarcinoma after curative-intent resection, even when matched for stage and nodal status [17]. Several non-exclusive hypotheses could explain this advantage: earlier symptomatic presentation (obstructive jaundice prompting timely diagnosis), anatomical containment that limits early perineural or vascular spread, and distinct evolutionary constraints at the ampullary interface that temper the lethal phenotypes seen in PDAC. Irrespective of the underlying mechanism, these observations reinforce the central message that AC warrants disease-specific pathways, not simple assimilation into duodenal, pancreatic, or biliary paradigms.

Therapeutically, the lineage-first, mutation-fast pathway articulated here provides a workable scaffold. Front-line therapy should be histology-directed (colorectal backbones for intestinal disease; pancreaticobiliary backbones for pancreatobiliary or mixed disease). At the earliest progression, profile fast and broadly (DNA + RNA) to capture HER2, MSI-H/dMMR, BRAF V600E, NTRK/RET fusions, and KRAS class, then pivot to matched therapies or clinical trials. The targeted-therapy signal specifically documented in AC remains limited but real, while tissue-agnostic approvals for NTRK and RET fusions and the growing HER2 experience in biliary cohorts create credible avenues for biomarker-defined subsets. KRAS p.G12C is rare in AC, but as allele-selective KRAS G12D and pan-RAS inhibitors mature in PDAC [95], AC should be prospectively included in expansion cohorts, with outcomes reported by lineage.

Beyond single-layer genomics, detailed multi-omic and proteogenomic interrogation can now refine both therapeutic design and prognostic stratification. Integrative analyses of DNA, RNA, and protein have identified metabolic- and adhesion-driven clusters that transcend histologic boundaries, revealing pathway vulnerabilities, such as fatty-acid metabolism and focal-adhesion signaling, that may condition chemotherapy response and immunotherapy sensitivity [96]. Embedding these signatures into translational protocols could guide rational chemotherapy backbone selection, identify candidates for FAK or metabolic inhibitors, and inform composite biomarkers that outperform lineage alone. Multi-omic stratification could be used as a practical scaffold for next-generation trial design in this rare cancer.

Future progress depends less on single drugs than on fit-for-purpose trial architecture. Ampulla-enriched umbrella platforms embedded within biliary programs can stratify first-line therapy by lineage and open adaptive molecular cohorts at progression (HER2, MSI-H/dMMR, *BRAF* V600E, *NTRK*/*RET*, *KRAS* classes). Tumor-agnostic basket studies should pre-specify AC eligibility and mandate AC attribution in publications to end ambiguity about representation and benefit. Recognizing that AC is more than the intersection of two lineages, biologically distinct from right colon, duodenum, pancreas, and distal bile duct, provides the rationale for disease-specific subgroups, even within tumor-agnostic master protocols. Standardized histo-molecular reporting, timely access to sequencing, and deliberate inclusion of AC in next-wave HER2, RAS-pathway, fusion-targeted, and immunotherapy programs are the practical steps that will move the field from extrapolation to AC-specific therapy.

## Figures and Tables

**Table 1:** Key trials guiding current precision therapy in AC management.

Trial Identifier	Intervention	Target/Alteration	Patient Population	Outcomes	Reference
MyPathway	Trastuzumab + Pertuzumab	HER2 amplification/mutation	Metastatic HER2+ biliary tract cancers including AC	ORR 23%, DCR 51%, PFS 4.0 mo, OS 10.9 mo; KRAS WT had better outcomes	[43]
DESTINY-PanTumor02	Trastuzumab deruxtecan (T-DxD)	HER2-positive	Advanced HER2+ solid tumors including AC subset	ORR 37.1%, PFS 6.9 mo, OS 13.4 mo	[44]
Case report	Trastuzumab + chemotherapy	HER2 amplification	One metastatic HER2+ AC patient	Stable disease, some tumor shrinkage	[45]
(RC48-ADC)	RC48-ADC	HER2 overexpression	HER2+ BTC post-1L chemo	Phase II; Active, not recruiting	NCT04329429
DESTINY-BTC01	T-DxD + rilvegostomig	HER2-expressing	1L HER2+ BTC	Phase III; Recruiting	NCT06467357
KRYSTAL-1	Adagrasib	KRAS p.G12C	KRAS G12C solid tumors incl. GI cancers	ORR 35.1%, PFS 6.9 mo	[46]
NCT01943864	Trametinib MEK inhibitor	MAPK pathway alteration	Advanced BTC including AC	12-wk NPR 10%, SD 65%, 1 PR, one >120-wk response	[47]
BEAVER trial	Encorafenib + Binimetinib	Non-V600E BRAF mutations	Solid tumors incl. pancreaticobiliary	ORR 13%, PFS 2.4 mo; 1 AC with PR	[48]
ROAR trial	Dabrafenib + Trametinib	BRAF V600E mutation	Rare biliary tract cancers	ORR 53%, DoR 8.9 mo, PFS 9.0 mo, OS 13.5 mo	[49]
LOXO-TRK-14001, SCOUT, NAVIGATE	Larotrectinib	NTRK fusions	NTRK+ solid tumors	ORR 75–79%, PFS 28 mo, OS 44 mo, DoR >35 mo	[50]
ALKA-372-001, STARTRK-1, STARTRK-200.00.00 00:00:00	Entrectinib	NTRK fusions	NTRK+ solid tumors incl. cholangiocarcinoma	ORR 57%, PFS 11 mo	[51]
LIBRETTO-001	Selpercatinib	RET fusions	RET+ solid tumors incl. pancreatic/biliary	ORR 43.9%, DoR 24.5 mo, PFS 13.2 mo	[52]
KEYNOTE-158	Pembrolizumab	MSI-H/dMMR	MSI-H advanced solid tumors incl. AC	ORR 34.3%, DoR 22.7 mo	[53]
CheckMate 142	Nivolumab ± Ipilimumab	MSI-H/dMMR	Metastatic colorectal cancer	ORR up to 69% (front-line), durable	[54]
CheckMate 848	Nivolumab ± Ipilimumab	High TMB (≥10 mut/Mb)	Advanced TMB-H tumors	ORR combo 38.6% vs. mono 29.8%	[55]
NCT02715284	Dostarlimab	MSI-H/dMMR	MSI-H GI tumors	ORR 41.6%, durable	[56]

Summary of abbreviations: BRAF: v-Raf murine sarcoma viral oncogene homolog B; BTC: biliary tract cancer; CAPOX: capecitabine plus oxaliplatin; DoR: duration of response; dMMR: deficient mismatch repair; GI: gastrointestinal; HER2: human epidermal growth factor receptor 2; KRAS: Kirsten rat sarcoma viral oncogene homolog; MAPK: mitogen-activated protein kinase; mo: months; MSI-H: microsatellite instability-high; mut/Mb: mutations per megabase; NPR: non-progression rate; NTRK: neurotrophic tropomyosin receptor kinase; ORR: objective response rate; OS: overall survival; PFS: progression-free survival; PR: partial response; RET: rearranged during transfection; SD: stable disease; T-DxD: trastuzumab deruxtecan; TMB-H: tumor mutational burden-high; WT: wild-type.

## Data Availability

No new data were generated or analyzed in this study.

## Abbreviations

The following abbreviations are used in this manuscript:

AC	ampullary carcinoma
BTC	biliary tract cancer
BRAF	v-Raf murine sarcoma viral oncogene homolog B
cfDNA	cell-free DNA
ctDNA	circulating tumor DNA
dMMR	deficient mismatch repair
*ERBB2*/*HER2*	erb-b2 receptor tyrosine kinase 2/human epidermal growth factor receptor 2
ERCP	endoscopic retrograde cholangiopancreatography
EUS	endoscopic ultrasound
KRAS	Kirsten rat sarcoma viral oncogene homolog
MAPK	mitogen-activated protein kinase
MEK	mitogen-activated protein kinase
MSI-H	microsatellite instability-high
NGS	next-generation sequencing
NTRK	neurotrophic tyrosine receptor kinase
ORR	objective response rate
OS	overall survival
PD-1/PD-L1	programmed death-1/programmed death-ligand 1
PFS	progression-free survival
RET	rearranged during transfection
TMB	tumor mutational burden
